# Supplementary material for: AffyMAPSDetector: a software tool to characterize Affymetrix GeneChip™ expression arrays with respect to SNPs
Source: BMC Bioinformatics. 2007 Jul 30;8:276. doi: 10.1186/1471-2105-8-276 (PMC1959249; doi:10.1186/1471-2105-8-276)
Supplement: Additional file 13 — AffyMAPSDetector v1 source code. AffyMAPSDetector source code. [file 1471-2105-8-276-S13.zip › AffyMAPSDetector_v1_src/doc/affychip/snpdetector/util/PmMmMatrixLoader.html]

PmMmMatrixLoader


|  |  |  |  |
| --- | --- | --- | --- |
| **Overview** \| **Package** \| **Class** \| **Use** \| **Tree** \| **Deprecated** \| **Index** \| **Help** | | |  |
| **PREV CLASS**   NEXT CLASS | **FRAMES**    **NO FRAMES**     **All Classes** |  |  |
| SUMMARY: NESTED \| FIELD \| CONSTR \| METHOD | DETAIL: FIELD \| CONSTR \| METHOD |  |  |


---


## affychip.snpdetector.util Class PmMmMatrixLoader

```
java.lang.Object
  affychip.snpdetector.util.PmMmMatrixLoader
```

---

public class **PmMmMatrixLoader** extends java.lang.Object

Title: Probe SNP Detector

Description: Utility class to load (NxM) matrix corresponding to PM/MM
values, where, Probes/Genes are arranged row-wise, Samples/Experiments/Chips
are arranged column-wise, and each cell represents the PM/MM expresssion
value for a particular Probe/Gene in a particular Sample/Experiment. Once
the matrix is loaded, a two way mapping is build between probe(gene) and
sample(experiment/chip).

Copyright: Sunita Kumari -- Copyright (c) 2004

Company: George Mason University

**Version:**
:   1.1

**Author:**
:   Sunita Kumari

---

**Constructor Summary**

`PmMmMatrixLoader(java.io.File pmMmMatrixFile)`

`PmMmMatrixLoader(java.lang.String pmMmMatrixFilename)`


|  |  |
| --- | --- |
| **Method Summary** | |
| `java.util.HashMap` | `getGeneAnnot()` |
| `static void` | `main(java.lang.String[] args)` |
| `void` | `setGeneAnnot(java.util.HashMap geneAnnot)` |
| `void` | `setPmMmMatrixFile(java.io.File pmMmMatrixFile)`             This method loads the PM/MM ratio matrix file and builds the data model for the heatmap viewer. |
| `void` | `setPmMmMatrixFile(java.lang.String pmMmMatrixFilename)` |

**Methods inherited from class java.lang.Object**

`clone, equals, finalize, getClass, hashCode, notify, notifyAll, toString, wait, wait, wait`

**Constructor Detail**

### PmMmMatrixLoader

```
public PmMmMatrixLoader(java.lang.String pmMmMatrixFilename)
                 throws java.lang.Exception
```

---


### PmMmMatrixLoader

```
public PmMmMatrixLoader(java.io.File pmMmMatrixFile)
                 throws java.lang.Exception
```


**Method Detail**

### setPmMmMatrixFile

```
public void setPmMmMatrixFile(java.lang.String pmMmMatrixFilename)
                       throws java.lang.Exception
```

:   **Throws:**: `java.lang.Exception`

---


### setPmMmMatrixFile

```
public void setPmMmMatrixFile(java.io.File pmMmMatrixFile)
                       throws java.lang.Exception
```

:   This method loads the PM/MM ratio matrix file and builds the data model
    for the heatmap viewer.

    :   **Parameters:**: `pmMmMatrixFile` - type java.io.File - The PM/MM ratio matrix file. **Throws:**: `java.lang.Exception`

---


### main

```
public static void main(java.lang.String[] args)
```

---


### getGeneAnnot

```
public java.util.HashMap getGeneAnnot()
```

:   **Returns:**: Returns the geneAnnot.

---


### setGeneAnnot

```
public void setGeneAnnot(java.util.HashMap geneAnnot)
```

:   **Parameters:**: `geneAnnot` - The geneAnnot to set.


---


|  |  |  |  |
| --- | --- | --- | --- |
| **Overview** \| **Package** \| **Class** \| **Use** \| **Tree** \| **Deprecated** \| **Index** \| **Help** | | |  |
| **PREV CLASS**   NEXT CLASS | **FRAMES**    **NO FRAMES**     **All Classes** |  |  |
| SUMMARY: NESTED \| FIELD \| CONSTR \| METHOD | DETAIL: FIELD \| CONSTR \| METHOD |  |  |


---
